# Supplementary material for: RNA polymerase II pausing is essential during spermatogenesis for appropriate gene expression and completion of meiosis
Source: Nat Commun. 2024 Jan 29;15:848. doi: 10.1038/s41467-024-45177-3 (PMC10824759; doi:10.1038/s41467-024-45177-3)
Supplement: Supplementary file 1 — Supplementary Information [file 41467_2024_45177_MOESM1_ESM.pdf]

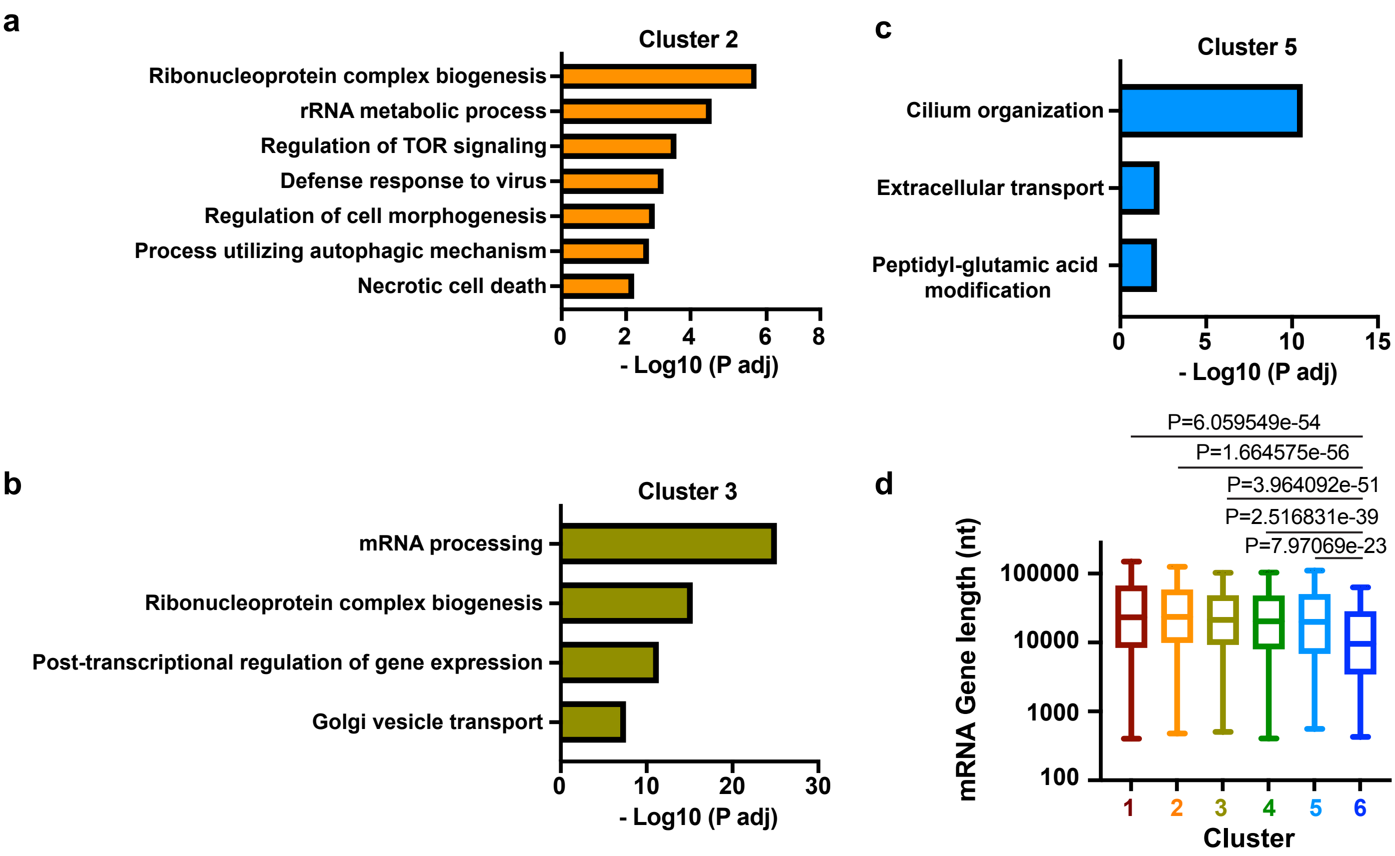

Supplementary Fig. 1. Gene ontology terms and mRNA gene lengths for clusters of differentially expressed genes.

a-c. Gene ontology terms and significance values for cluster 2 (**a**), cluster 3 (**b**), and cluster 5 (**c**) generated with ClusterProfiler and consolidated using Revigo.

d. Box plot representation of the distribution of gene length (TSS to TES) for protein-coding genes in each cluster. Cluster 6 mRNAs are significantly shorter than mRNAs in all other clusters, confirming that the abundance of lncRNAs in this cluster was not biasing the length determination shown in Fig. 1k. Aside from lncRNAs, no other class or family of genes was found to be enriched in cluster 6. Line represents median, box represents 25-75th percentile, whiskers represent 1.5X interquartile range. P-values are all from Mann Whitney U unpaired, two-sided test of cluster 1 (n=3,083), 2 (n=2,533), 3 (n=3,393), 4 (n=3,267), or 5 (n=1,239) compared to cluster 6 (n=1,197).

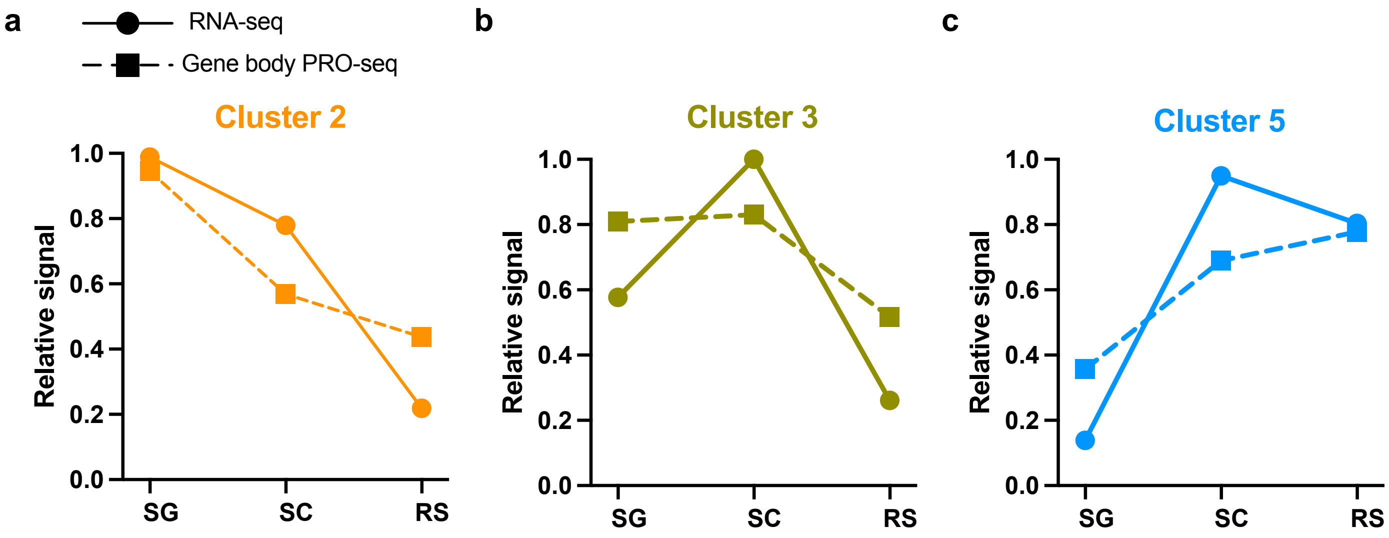

Supplementary Fig. 2. Relative signal from RNA and PRO-seq gene body across cell types.
a-c. Plots of average relative signal from RNA-seq (solid line and circle) or PRO-seq in gene bodies (dotted line and square) for each cell type, for clusters 2 (**a**), 3 (**b**), and 5 (**c**).

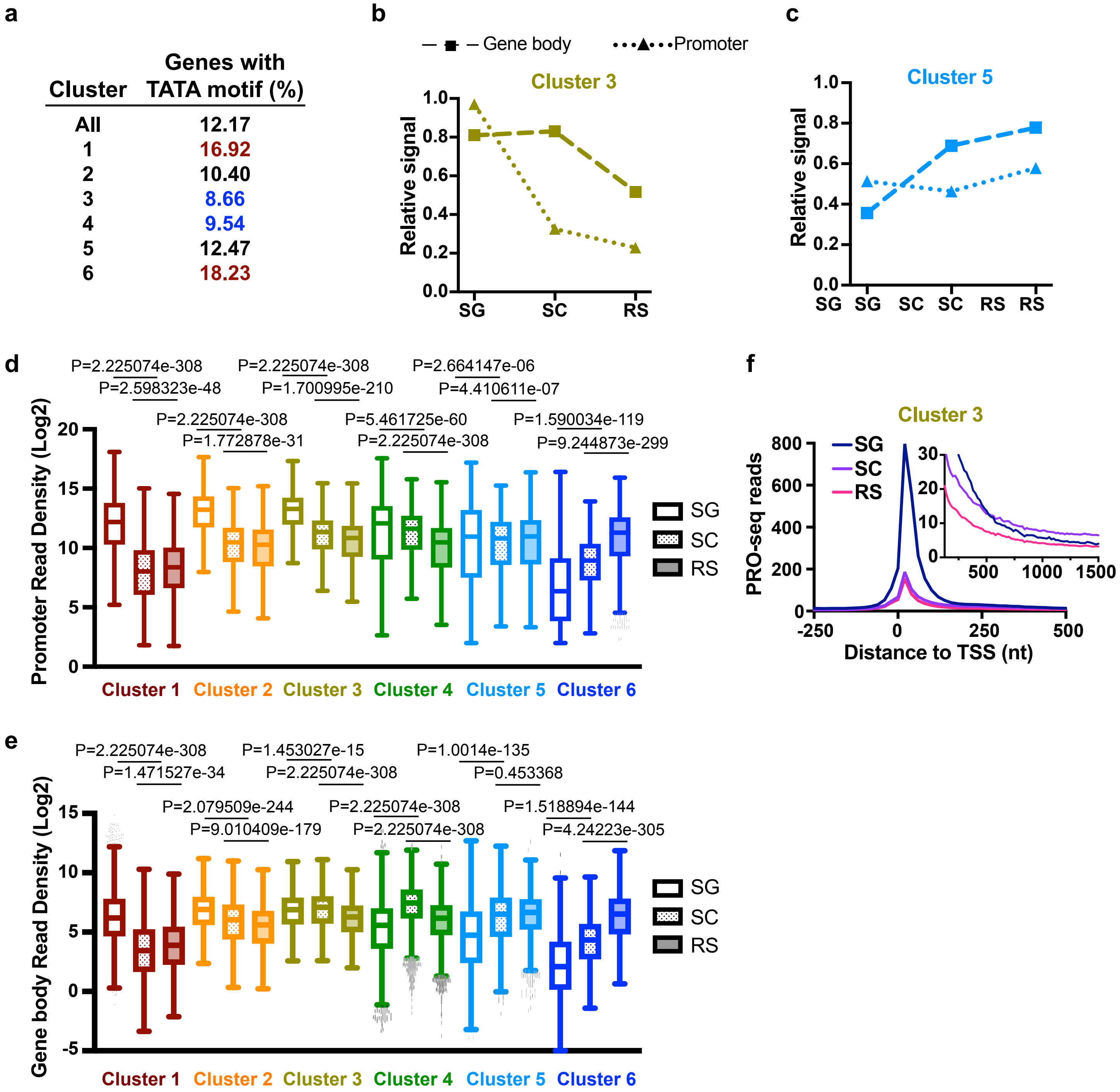

Supplementary Fig. 3 Promoter and gene body counts by cluster and cell type.
a. The proximal promoter regions (TSS-100 to TSS) for all clustered genes were evaluated for a match to the canonical TATA motif using FIMO with a threshold of P=0.005. The percent (%) of genes in each individual cluster is also reported. Clusters 1 and 6 have an enrichment of TATA elements, whereas Clusters 3 and 4 are depleted of TATA motifs.
b-c. Plots of average relative signal from PRO-seq within promoter (dotted line with triangles) or gene body (dashed line with squares) regions for each cell type. Shown are data for genes in clusters 3 (**b**) 5 (**c**).
d-e. Box plot representations for the distribution of reads per kb in each cluster from PRO-seq promoter, (TSS to +150, **d**) or gene body (TSS+250 to TES, **e**), for each cell type. Legend indicates SG (empty), SC (dotted), and RS (shaded). P-values are from two-sided Wilcoxon matched-pairs test.
f. Metagene plot of average PRO-seq reads in each cell type for all protein-coding genes in cluster 3. Inset y-axis is zoomed-in to show gene body signal from +125 to +1500 nt downstream of the TSS.

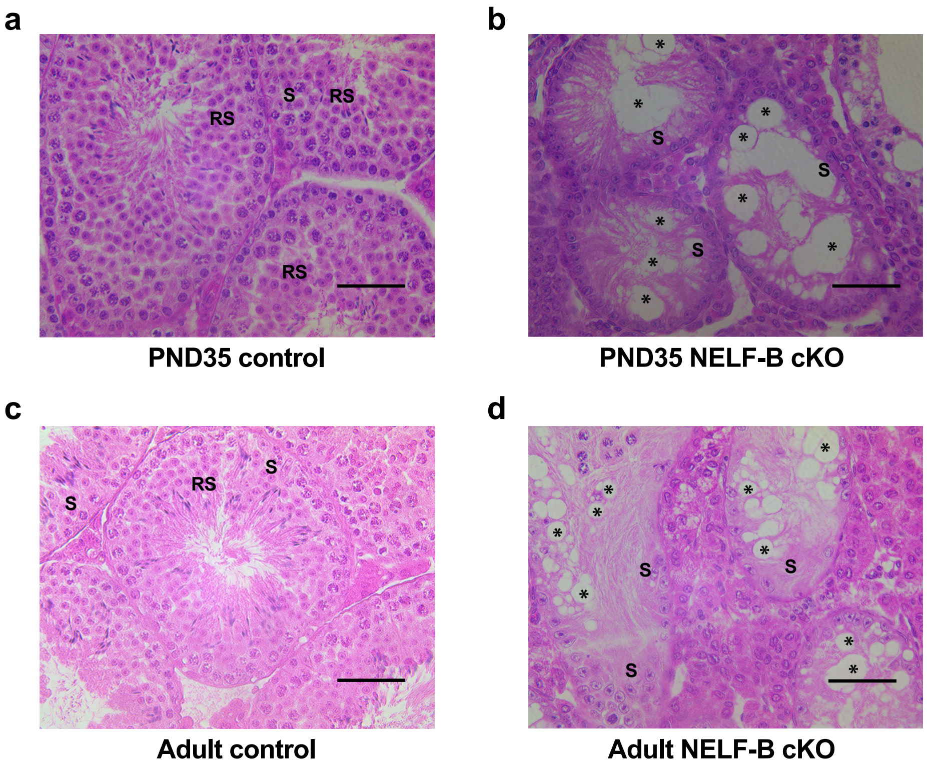

Supplementary Fig. 4. NELF-B is required for the successful completion of spermatogenesis.
a-b. H & E staining of testis cross sections of PND35 control (**a**) or NELF-B cKO (**b**) mice. NELF-B cKO testis show severe degeneration of the epithelium with vacuoles (asterisk) and only Sertoli cells (S) remaining in the epithelium compared to control mice which successfully generate round spermatids (RS). Scale bar, 50 µm.
c-d. H & E staining of testis cross sections of adult 9-month old control (**c**) or NELF-B cKO (**d**) mice. There was no recovery of spermatogenesis in the adult as shown by the vacuoles (asterisk) and lack of germ cells progressing to RS. Scale bar, 50 µm.

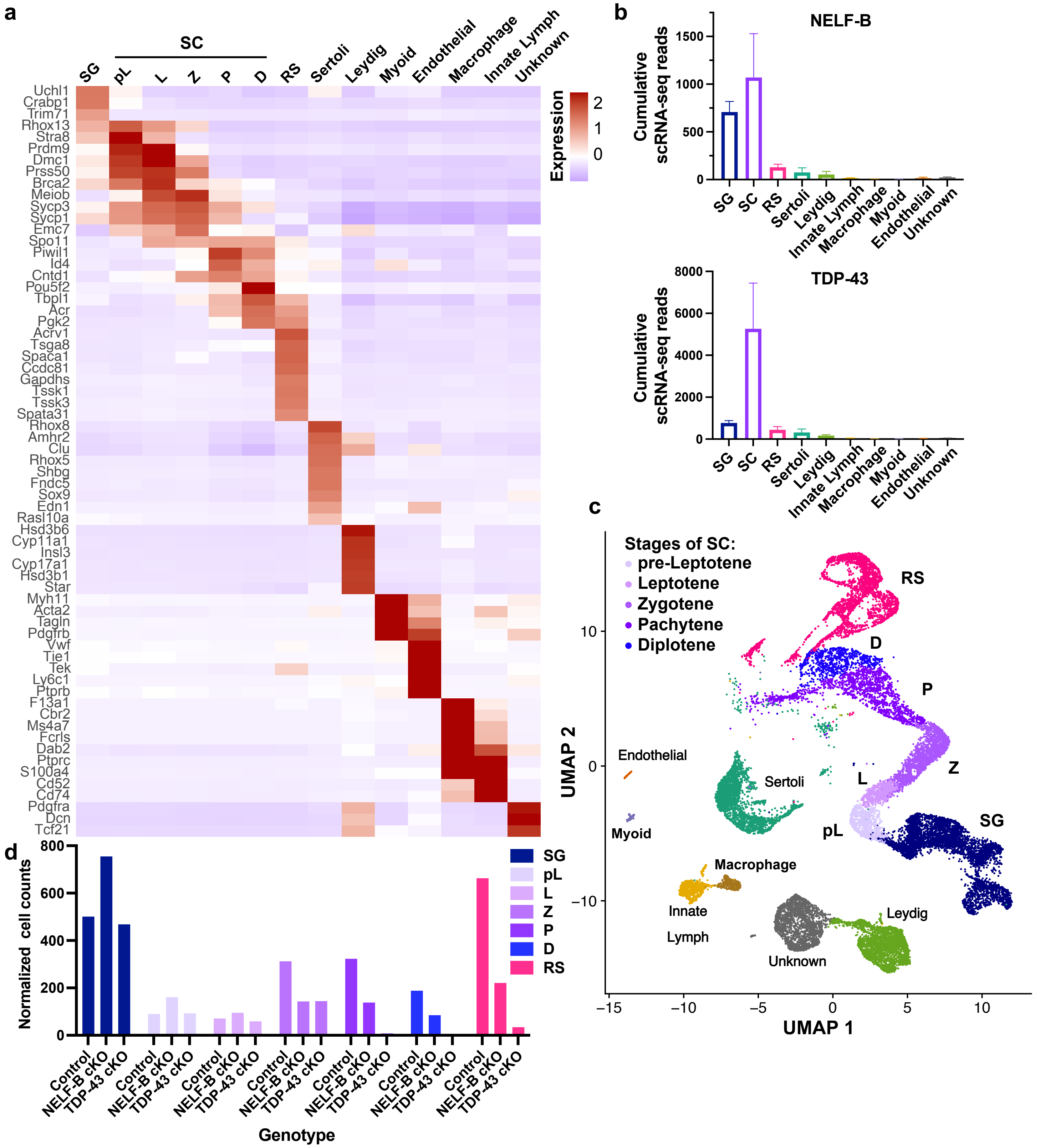

Supplementary Fig. 5 Marker genes and further characterization of scRNA-seq clusters.

a. Marker gene expression from scRNA-seq in control mice (n=4). Shown at left are the marker genes used to assign clusters to specific cell types. For this heatmap, signal for each gene was normalized within the row, to compare expression across cell types.

b. Gene expression of NELF-B (left) or TDP-43 (right) in control mice (n=4) for the indicated cell type. Shown are the mean and standard deviation of cumulative counts from scRNA-seq for each cell type.

c. UMAP representation of all 14 somatic and germ cell type clusters including the stages of SC, integrated across the 3 genotypes (n=24,269 cells). Normalized cell counts in control samples (SG=501, pre-Leptotene=90, Leptotene=71, Zygotene=312, Pachytene=323, Diplotene=188, RS=663).

d. Numbers of each germ cell type (including stages of SC) per genotype. Cell counts were normalized by the number of mice per genotype (Control n=4, NELF-B cKO n=2, TDP-43 cKO n=3).

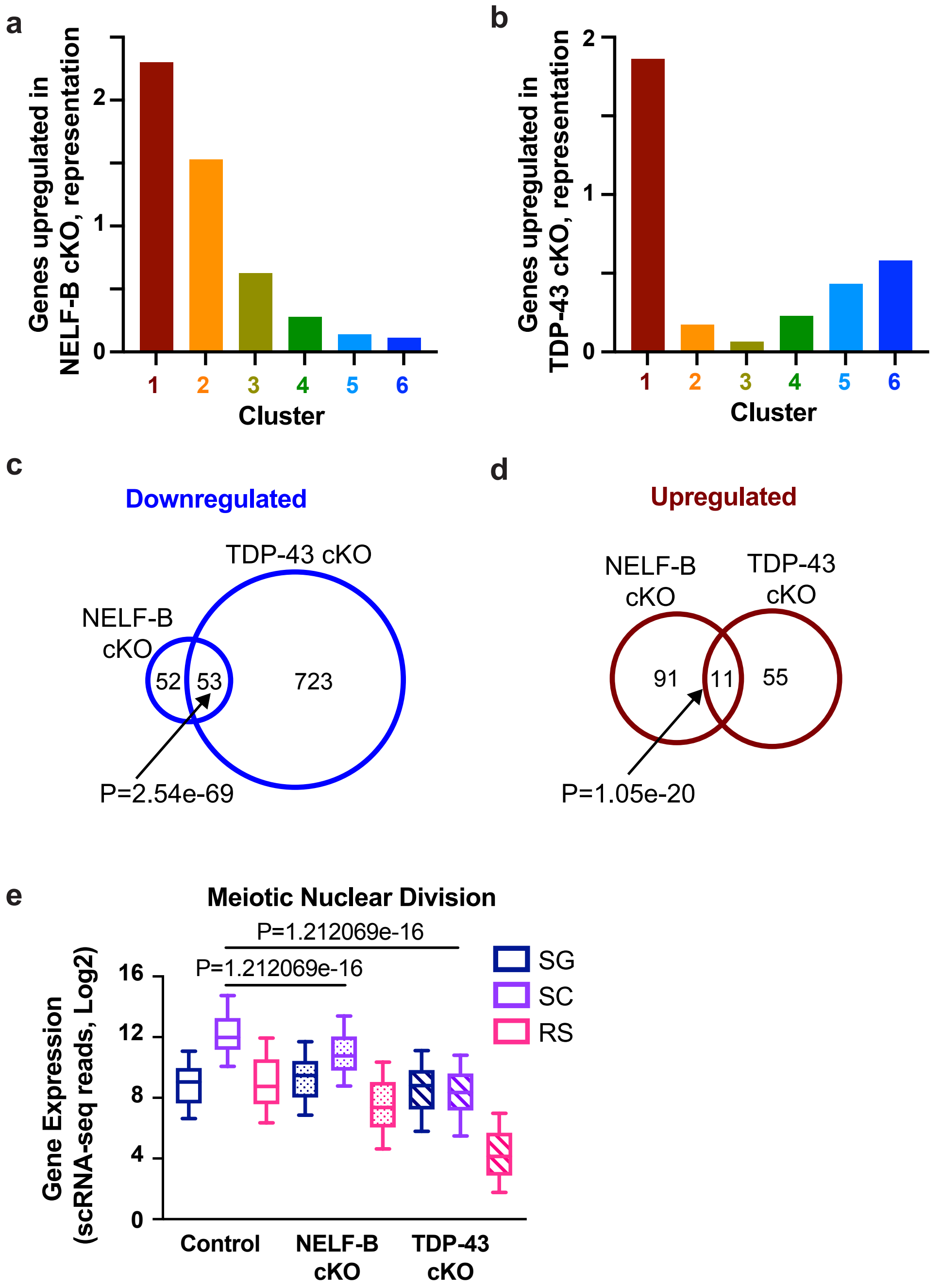

Supplementary Fig. 6 Investigation of significantly differentially expressed genes in NELF-B cKO and TDP-43 cKO mice.

a-b. Representation of upregulated genes from NELF-B cKO (**a**) or TDP-43 cKO mice (**b**), within each cluster (from Fig. 1f). A value of 1 indicates representation expected by chance.

c-d. Overlap between genes downregulated (**c**) or upregulated (**d**) in NELF-B and TDP-43 mice. P-value for the overlap of gene lists was calculated using the hypergeometric distribution with the phyper function in R

e. Box plots show distribution of expression in scRNA-seq for all 91 Cluster 4 genes in the Gene Ontology category Meiotic Nuclear Division. Expression is shown from control, NELF-B cKO, or TDP-43 cKO mice in SG, SC, or RS cells, as indicated. Line represents median, box represents 25-75th percentile, whiskers represent 10-90th percentile. P-values from two-sided Wilcoxon matched pairs test.

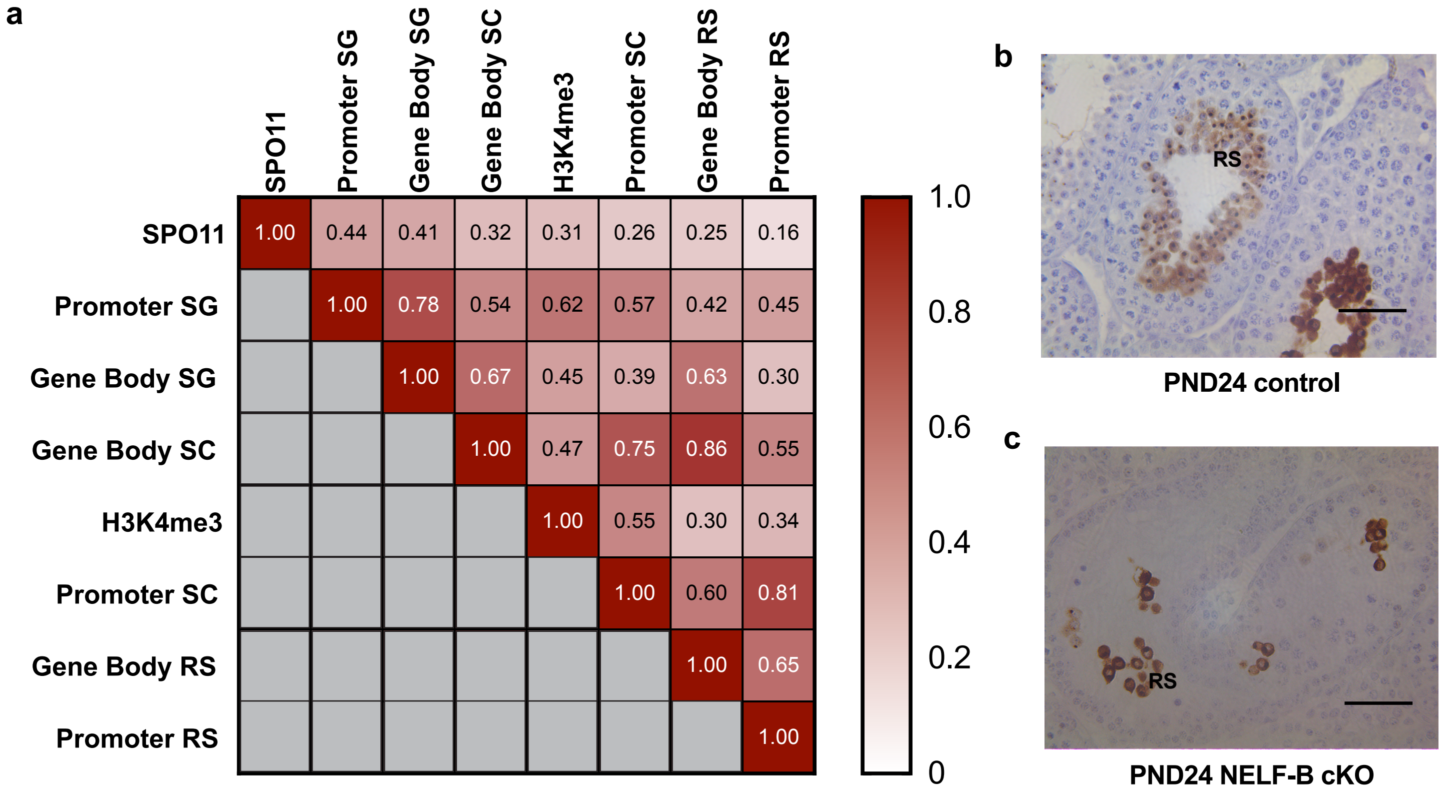

Supplementary Fig. 7. SPO11 oligo reads indicative of DSBs correlate best with promoter PRO-seq signal in SG, and NELF-B cKO mice generate very few post-meiotic germ cells.

a. Heatmap matrix of Spearman's correlation comparisons between SC SPO11 oligo sequencing (sum TSS+-500), H3K4me3 ChIP-seq (Sum TSS+-500), and PRO-seq promoter and gene body windows in SG, SC, and RS across all DE genes (n=17,078). Data sets are ordered left to right / top to bottom based on correlation with SPO11.

b-c. Immunohistochemistry of PND24 mouse testis using an antibody to the acrosomal marker SP-10. Control PND24 testis show the luminally arranged post-meiotic round spermatids (RS) with acrosome stained (**b**). PND24 NELF-B cKO testis show very few antibody-positive RS, indicating meiotic failure (**c**). Scale bar, 50 μm.

<u>**Supplementary Table 1: List of meiotic nuclear division genes from Cluster 4**</u>

Gene name	Description
Hsf2bp	Heat shock transcription factor 2 binding protein
Msh4	Muts homolog 4
Spo11	Spo11 initiator of meiotic double stranded breaks
Cdc20	Cell division cycle 20
Msh5	Muts homolog 5
Rad51c	Rad51 paralog c
Spire2	Spire type actin nucleation factor 2
Tex14	Testis expressed gene 14
Asz1	Ankyrin repeat, sam and basic leucine zipper domain containing 1
Dmrtc2	Doublesex and mab-3 related transcription factor like family c2
Chfr	Checkpoint with forkhead and ring finger domains
Sirt2	Sirtuin 2
Mov10l1	Mov10 like risc complex rna helicase 1
Birc5	Baculoviral iap repeat-containing 5
Psmc3ip	Proteasome (prosome, macropain) 26s subunit, atpase 3, interacting protein
Ubb	Ubiquitin b
Sycp3	Synaptonemal complex protein 3
Fzr1	Fizzy and cell division cycle 20 related 1
Meikin	Meiotic kinetochore factor
Pttg1	Pituitary tumor-transforming gene 1
Hormad2	Horma domain containing 2
4930447C04Rik	Riken cdna 4930447c04 gene
Mlh3	Mutl homolog 3
Ddx4	Dead box helicase 4
Rad1	Rad1 checkpoint dna exonuclease
Ercc4	Excision repair cross-complementing rodent repair deficiency, complementation group 4
Meiob	Meiosis specific with ob domains
Bag6	Bcl2-associated athanogene 6
Majin	Membrane anchored junction protein
Mus81	Mus81 structure-specific endonuclease subunit
Cenpx	Centromere protein x
Syce1	Synaptonemal complex central element protein 1
Mybl1	Myeloblastosis oncogene-like 1
Sgo2a	Shugoshin 2a
Nuf2	Nuf2, ndc80 kinetochore complex component
Terb2	Telomere repeat binding bouquet formation protein 2
Aurka	Aurora kinase a
Sycp1	Synaptonemal complex protein 1
Hormad1	Horma domain containing 1
Ccne2	Cyclin e2
Eif4g3	Eukaryotic translation initiation factor 4 gamma, 3
Cenpc1	Centromere protein c1
Brdt	Bromodomain, testis-specific
Rad51ap1	Rad51 associated protein 1
Tdrd12	Tudor domain containing 12

Mre11a	Mre11a homolog a, double strand break repair nuclease
Tex12	Testis expressed 12
Ccnb2	Cyclin b2
Cep63	Centrosomal protein 63
Syce1l	Synaptonemal complex central element protein 1 like
Piwil2	Piwi-like rna-mediated gene silencing 2
Fancd2	Fanconi anemia, complementation group d2
Smc4	Structural maintenance of chromosomes 4
Rmi1	Recq mediated genome instability 1
Fignl1	Fidgetin-like 1
Psma8	Proteasome subunit alpha 8
Siah1a	Siah e3 ubiquitin protein ligase 1a
Stag3	Stromal antigen 3
Zscan21	Zinc finger and scan domain containing 21
Ttk	Ttk protein kinase
Tex19.2	Testis expressed gene 19.2
Slx4	Slx4 structure-specific endonuclease subunit homolog (s. Cerevisiae)
Mael	Maelstrom spermatogenic transposon silencer
Brca2	Breast cancer 2, early onset
Tdrkh	Tudor and kh domain containing protein
Ago4	Argonaute risc catalytic subunit 4
Shcbp1l	Shc sh2-domain binding protein 1-like
Hfm1	Hfm1, atp-dependent dna helicase homolog
Mcmdc2	Minichromosome maintenance domain containing 2
Npm2	Nucleophosmin/nucleoplasmin 2
Fbxo43	F-box protein 43
Morc2b	Microrchidia 2b
Catsperz	Cation channel sperm associated auxiliary subunit zeta
Plcb1	Phospholipase c, beta 1
Meioc	Meiosis specific with coiled-coil domain
Spdya	Speedy/ringo cell cycle regulator family, member a
Terb1	Telomere repeat binding bouquet formation protein 1
Tdrd9	Tudor domain containing 9
Rnf212	Ring finger protein 212
Espl1	Extra spindle pole bodies 1, separase
Hspa2	Heat shock protein 2
Sycp2	Synaptonemal complex protein 2
Cks2	Cdc28 protein kinase regulatory subunit 2
Mapk15	Mitogen-activated protein kinase 15
Ccnb1ip1	Cyclin b1 interacting protein 1
Eme2	Essential meiotic structure-specific endonuclease subunit 2
Cntd1	Cyclin n-terminal domain containing 1
Rad54b	Rad54 homolog b (s. Cerevisiae)
1700028K03Rik	Riken cdna 1700028k03 gene
Ankrd31	Ankyrin repeat domain 31
Spata22	Spermatogenesis associated 22
