# Supplementary material for: RNA polymerase II pausing is essential during spermatogenesis for appropriate gene expression and completion of meiosis
Source: Nat Commun. 2024 Jan 29;15:848. doi: 10.1038/s41467-024-45177-3 (PMC10824759; doi:10.1038/s41467-024-45177-3)
Supplement: Supplementary file 3 — Reporting Summary [file 41467_2024_45177_MOESM3_ESM.pdf]

nature portfolio

Corresponding author(s): Dr. Karen Adelman and Dr. Prabhakara Reddi

Last updated by author(s): Dec 21, 2023

Reporting Summary

Nature Portfolio wishes to improve the reproducibility of the work that we publish. This form provides structure for consistency and transparency in reporting. For further information on Nature Portfolio policies, see our Editorial Policies and the Editorial Policy Checklist.

Statistics

For all statistical analyses, confirm that the following items are present in the figure legend, table legend, main text, or Methods section.

n/a	Confirmed	
☐	☒	The exact sample size (*n*) for each experimental group/condition, given as a discrete number and unit of measurement
☐	☒	A statement on whether measurements were taken from distinct samples or whether the same sample was measured repeatedly
☐	☒	The statistical test(s) used AND whether they are one- or two-sided<br>*Only common tests should be described solely by name; describe more complex techniques in the Methods section.*
☒	☐	A description of all covariates tested
☐	☒	A description of any assumptions or corrections, such as tests of normality and adjustment for multiple comparisons
☐	☒	A full description of the statistical parameters including central tendency (e.g. means) or other basic estimates (e.g. regression coefficient) AND variation (e.g. standard deviation) or associated estimates of uncertainty (e.g. confidence intervals)
☐	☒	For null hypothesis testing, the test statistic (e.g. *F*, *t*, *r*) with confidence intervals, effect sizes, degrees of freedom and *P* value noted<br>*Give P values as exact values whenever suitable.*
☒	☐	For Bayesian analysis, information on the choice of priors and Markov chain Monte Carlo settings
☒	☐	For hierarchical and complex designs, identification of the appropriate level for tests and full reporting of outcomes
☐	☒	Estimates of effect sizes (e.g. Cohen's *d*, Pearson's *r*), indicating how they were calculated

Our web collection on statistics for biologists contains articles on many of the points above.

Software and code

Policy information about availability of computer code

Data collection	NGS: Illumina platforms as indicated for each sample on GEO with appropriate Illumina Realtime Analysis, base calling and demultiplexing software as used by HMS Bauer core or Novogene.<br>For the 10x Genomics scRNA-seq, base calling and demultiplexing was performed by the DNA Services laboratory of the Roy J. Carver Biotechnology Center at the University of Illinois at Urbana-Champaign.
Data analysis	For RNA-seq data mapping and analysis: STAR v2.7.3a, DESeq2 v1.36.0.<br>For PRO-seq data mapping and analysis: cutadapt v1.14, bowtie2 v2.3.4.3, samtools v1.3.1, R v3.6.1<br>For scRNA-seq data mapping and analysis: 10x Genomics Cell Ranger 7.0.0, Seurat v4.2.0<br>For gene ontology categories: clusterProfiler v4.4.4was used.<br><br>The data tables generated in R version 3.6.1 were copied into Graphpad Prism v9.5.1 or Partek Genomics Suite v7.19.1125 for plotting and statistical tests.<br><br>No new scripts were generated for this study. Custom scripts including make_heatmap, bowtie2stdBedGraph.pl, and normalize_bedGraph are all publicly available (https://github.com/AdelmanLab/NIH_scripts; https://doi.org/10.5281/zenodo.5519914). The Get Gene Annotation script is publicly available (https:// github.com/AdelmanLab/GetGeneAnnotation_GGA; https://doi.org/10.5281/zenodo.5519927). Additionally, the scRNA-seq tutorial from the Harvard Chan Bioinformatics Core is publicly available (https://doi.org/10.5281/zenodo.5826256).

For manuscripts utilizing custom algorithms or software that are central to the research but not yet described in published literature, software must be made available to editors and reviewers. We strongly encourage code deposition in a community repository (e.g. GitHub). See the Nature Portfolio guidelines for submitting code & software for further information.

Data

Policy information about availability of data

All manuscripts must include a data availability statement. This statement should provide the following information, where applicable:

- Accession codes, unique identifiers, or web links for publicly available datasets
- A description of any restrictions on data availability
- For clinical datasets or third party data, please ensure that the statement adheres to our policy

Bulk RNA-seq, PRO-seq, and scRNA-seq data generated in this study were deposited in the NCBI GEO database under accession code GSE228454 (https://www.ncbi.nlm.nih.gov/geo/query/acc.cgi?acc=GSE228454). Previously published SPO11 oligo data is available at the NCBI GEO database under accession code GSE84689 (https://www.ncbi.nlm.nih.gov/geo/query/acc.cgi?acc=GSE84689). Published H3K4me3 ChIP-seq data is available at the NCBI GEO database under accession code GSE52628 (https://www.ncbi.nlm.nih.gov/geo/query/acc.cgi?acc=GSE52628). The mouse reference genome mm10 is publicly available from UCSC https://hgdownload.soe.ucsc.edu/goldenPath/mm10/bigZips/.

Research involving human participants, their data, or biological material

Policy information about studies with human participants or human data. See also policy information about sex, gender (identity/presentation), and sexual orientation and race, ethnicity and racism.

Reporting on sex and gender	N/A
Reporting on race, ethnicity, or other socially relevant groupings	N/A
Population characteristics	N/A
Recruitment	N/A
Ethics oversight	N/A

Note that full information on the approval of the study protocol must also be provided in the manuscript.

Field-specific reporting

Please select the one below that is the best fit for your research. If you are not sure, read the appropriate sections before making your selection.

☒ Life sciences ☐ Behavioural & social sciences ☐ Ecological, evolutionary & environmental sciences

For a reference copy of the document with all sections, see nature.com/documents/nr-reporting-summary-flat.pdf

Life sciences study design

All studies must disclose on these points even when the disclosure is negative.

Sample size	In determining sample sizes, we made all possible efforts to minimize animal suffering. To isolate spermatogonia, spermatocytes, and round spermatids for PRO-seq and bulk RNA-seq experiments the number of wild type male C57BL/6J mice was determined based on the number of germ cells required for PRO-seq and RNA-seq. Where conditional knockout (cKO) mice were used for single cell RNA-seq we were mindful of the cost, time, and difficulty obtaining mutant mice of the required genotype. For the single cell RNA-seq, histology, and immunohistochemistry, and immunofluorescence experiments, n=3 was used for experimental and control mice except as noted. The results were similar between biological replicates. No statistical method was used to predetermine sample size.<br>For RNA-seq and PRO-seq experiments, 3 biological replicates were performed for each cell type. Cells were isolated and pooled from 25 mice (spermatogonia) or 10 mice (spermatocytes and round spermatids). The number of mice was selected in order to generate enough material for 1-2 million cells per replicate of each cell type for each experiment.<br>For scRNA-seq, sample size was based on successful generation of conditional knockout NELF-B or TDP-43 mice. For NELF-B cKO, n=2, TDP-43 cKO, n=3. Litter mate controls were used for comparison, n=4.
Data exclusions	No data was excluded, all data filtering is described in the methods and figure legends.
Replication	Principal component analysis was used to confirm clustering of replicates within each cell type for RNA-seq and PRO-seq. PRO-seq replicates were further evaluated for agreement using Spearman's r correlation of promoter counts TSS to +150. All were r = .95 or higher.<br>Experiments were performed using a minimum of 3 biological replicates which provided similar results. For our scRNAseq experiment, because of the cost involved and the difficulty in obtaining knockout mice we used 2 biological replicates for the NELF-B cKO scRNAseq which also gave similar results.
Randomization	Randomization was not used in this study. Where conditions are compared (e.g. control versus conditional knockout), samples were always processed in parallel to eliminate batch effects.

Blinding	Blinding was not used in this study. This is not a clinical research study and no human participants were involved.

Reporting for specific materials, systems and methods

We require information from authors about some types of materials, experimental systems and methods used in many studies. Here, indicate whether each material, system or method listed is relevant to your study. If you are not sure if a list item applies to your research, read the appropriate section before selecting a response.

Materials & experimental systems

n/a	Involved in the study
☐	☒ Antibodies
☒	☐ Eukaryotic cell lines
☒	☐ Palaeontology and archaeology
☐	☒ Animals and other organisms
☒	☐ Clinical data
☒	☐ Dual use research of concern
☒	☐ Plants

Methods

n/a	Involved in the study
☒	☐ ChIP-seq
☒	☐ Flow cytometry
☒	☐ MRI-based neuroimaging

Antibodies

Antibodies used	1. NELFB Rabbit Polyclonal antibody from Proteintech Cat No. 16418-1-AP (1:100 dilution)<br>2. SP-10 Guinea Pig Polyclonal antibody (In-house antibody) made by the Reddi lab; Osuru et al., Mol. Reprod. Dev 81:896-907 (2014) (1:1000 dilution)<br>3. SYCP3 RAbbit Polyclonal Antibody from Abcam (15093) (1:200 dilution)<br>4. γH2AX Mouse Monoclonal Antibody from Millipore Sigma (05-636) (1:500 dilution)<br>5. RPA Rabbit Monoclonal Antibody from Abcam (76420) (1:200 dilution)<br>6. HRP-conjugated anti-Rabbit secondary antibody (Jackson Immunoresearch Laboratories, Code Number: 111-035-144) at a 1:200 dilution<br>7. Peroxidase-conjugated AffiniPure Donkey Anti-Guinea Pig IgG (H+L) (Jackson ImmunoResearch Laboratories, Code Number: 706-035-148) at a 1:200 dilution<br>8. Goat secondary antibodies (anti-mouse 594 [Molecular Probes; A11020; 1:1000 dilution])<br>9. Anti-rabbit 488 [Molecular Probes; A11070; 1:1000 dilution])
Validation	1. NELFB antibody was affinity purified by Proteintech. Validated by Proteintech by knockdown in Hela cells. Further validation came from the present study in which the antibody did not react with NELF-B cKO mouse testis germ cells.<br>2. SP-10 antibody validated by immunohistochemistry of mouse testis cross sections showing staining of the acrosome of round spermatids. Osuru et al., Mol. Reprod. Dev 81:896-907 (2014)<br>3. SYCP3 antibody is immunogen affinity purified and therefore highly specific. Further, Abcam validated it by showing immunoreactivity with the Synaptonemal complex of mouse spermatocytes.<br>4. γH2AX antibody is immunoaffinity purified and validated by Millipore Sigma by Chromatin-IP and Western blotting<br>5. RPA antibody is Protein A purified and validated by Abcam by showing immunoreactivity with expected band size (29 kDa) in multiple cell lines.

Animals and other research organisms

Policy information about studies involving animals; ARRIVE guidelines recommended for reporting animal research, and Sex and Gender in Research

Laboratory animals	Mice were used in the study. Mus musculus C57BL/6J, males aged 6-8 days, 15 days, 24 days, 35 days, 3 months, and 9 months. Mice were maintained in breeding cages at a constant temperature of 21°C with 50-60% humidity under a 12 hour dark and 12 hour light cycle. Food and water were made available ad libitum.
Wild animals	No wild animals were used in the study.
Reporting on sex	Findings apply only to male mice. Because the study was on testicular spermatogenic cells, only males were used. Female mice could not be used. Male sex was assigned based on shorter anogenital distance. There is no sex-based analysis because only male mice were used for the study, which involves testicular germ cells. Female mice were not used.
Field-collected samples	No field collected samples were used in the study.
Ethics oversight	Institutional Animal Care and Use Committee (IACUC) of the University of Illinois Urbana Champaign (UIUC) approved the animal use protocol (21206) entitled, "Animal models to study spermatogenesis and male infertility".

Note that full information on the approval of the study protocol must also be provided in the manuscript.
